# NDRG4, a novel candidate tumor suppressor, is a predictor of overall survival of colorectal cancer patients

**DOI:** 10.18632/oncotarget.3170

**Published:** 2015-02-05

**Authors:** Dake Chu, Zixi Zhang, Yi Zhou, Yunming Li, Shaojun Zhu, Jian Zhang, Qingchuan Zhao, Gang Ji, Weizhong Wang, Jianyong Zheng

**Affiliations:** ^1^ State Key Laboratory of Cancer Biology and Xijing Hospital of Digestive Diseases, Xijing Hospital, Fourth Military Medical University, Xi'an, China; ^2^ State Key Laboratory of Cancer Biology, Department of Biochemistry and Molecular Biology, Fourth Military Medical University, Xi'an, China; ^3^ Department of Plastic Surgery, Xijing Hospital, Fourth Military Medical University, Xi'an, China; ^4^ Department of Gastrointestinal Surgery, Tianjin Union Medical Center, Tianjin, China; ^5^ Statistics Office, Chengdu Military General Hospital, Chengdu, Sichuan Province, China; ^6^ Department of Pathology, Fourth Military Medical University, Xi'an, China

**Keywords:** NDRG4, PI3K-AKT, colorectal cancer, carcinogenesis, progression

## Abstract

The role of NDRG4 in human malignancies is largely unknown. We investigated the role of NDRG4 protein in colorectal cancer and its prognostic value in a hospital-based retrospective training cohort of 272 patients and a prospective validation cohort of 708 patients were. Cell line was transfected with an NDRG4 expression construct to confirm the suppression of PI3K-AKT activity by NDRG4. Appropriate statistical methods were utilized for analysis. Results showed that NDRG4 protein expression was significantly decreased from normal mucosa, chronic colitis, ulcerative colitis, atypical hyperplasia to colorectal cancer. Significant negative correlations were found between NDRG4 staining and p-AKT. Patients with positive NDRG4 staining had favorable survival in both study cohorts. In multivariate analysis, NDRG4 staining proved to be an independent predictor of overall survival. Moreover, the prognostic role of NDRG4 was stratified by p-AKT. Overexpression of NDRG4 in colorectal cancer cell can significantly suppress PI3K-AKT activity, even after EGF stimulation. These results indicated NDRG4 protein expression was decreased in colorectal cancer. It may play its tumor suppressive role in carcinogenesis and progression through attenuation of PI3K-AKT activity. Therefore, high risk colorectal cancer patients could be better identified based on the combination of NDRG4 and PI3K-AKT activity.

## INTRODUCTION

N-Myc downstream-regulated gene 4 (NDRG4) is a member of NDRG family, which comprises four members named NDRG1–4 that have 57%–65% amino acid sequence homology [1, 2]. These four members can be divided into two subfamilies based on sequence homology: NDRG1 and NDRG3 are in one subfamily while NDRG2 and NDRG4 constitute another [3, 4]. NDRG4, a new tumor suppressor candidate gene, is located at chromosome 16q21–q22.3, spans 26 kilobases, and contains 17 exons [1, 5]. In contrast to other NDRG members, previous study found that NDRG4 was specifically expressed in brain and heart [1, 6–8]. It was reported that NDRG4 expression was down-regulated under severe ventricular hypoplasia in embryonic mouse heart, indicating its role in cell growth and proliferation [9]. In mouse brain, NDRG4 was identified in neuronal cytoplasm of cerebrum and cerebellum [10]. However, a recent study found that the prevalence of NDRG4 promoter methylation was increased in colorectal cancer [11]. This promoter methylation pattern suggested that NDRG4 might play an important role within colorectal cancer carcinogenesis and progression.

However, to our knowledge, the protein expression of NDRG4 and its role in tumor carcinogenesis, progression and prognosis has not been addressed yet. And the potential mechanism behind the function of NDRG4 was still unclear. We initially cloned human NDRG2 (GenBank^TM^ No. AF159092) and found it is associated with tumor differentiation, progression and prognosis in colorectal cancer [12–14]. Accumulating evidence then suggested that NDRG2 played a tumor suppressor role in various human malignancies [15–20]. As NDRG4 is more than 60% identical in amino acid sequence to NDRG2, this sequence similarity suggests NDRG4 may recapitulate the tumor suppressive function of NDRG2 in colorectal cancer. The carcinogenesis of colorectal cancer represent a distinguishing feature different from other malignancies, as increasing evidences showed the close relation between colorectal cancer and chronic intestinal inflammation may lead the development of colorectal cancer [21, 22]. Thus, inflammation related signals might be the target of NDRG4 during colorectal cancer carcinogenesis and progression. Phosphatidylinositol 3-kinase (PI3K)-AKT signaling pathway, one of the major inflammation and oncogenic pathways, is frequently activated in colorectal colitis and tumorigenesis [23, 24]. Its activation has been considered to be the most crucial factor in the pathogenesis of colorectal inflammation and its transformation to cancer [25, 26]. Thus, we speculate that NDRG4 might possess a tumor suppressor role in tumor carcinogenesis, invasion and metastasis of colorectal cancer by its interaction with PI3K-AKT signal.

In the present study, we investigated the NDRG4 protein expression and its role in colorectal cancer carcinogenesis, progression and prognosis, as well the association of NDRG4 with PI3K-AKT activity.

## RESULTS

### Characteristics of patients and specimens

The characteristics of the 980 colorectal cancer patients involved in the study cohort are showed in Table 1. 460 (46.9%) were female and 520 (53.1%) were male. 562 (57.3%) cases were less than 60 years old while 418 (42.7%) cases were over 60. Among all specimens recruited, 221 tumors (22.6%) were located in right colon, 280 tumors (28.6%) were located in left colon and 479 (48.9%) were located in rectum. The tumor size of 225 colorectal cancer (23.0%) was smaller than 3 cm (including 3 cm) and that of 755 (77.0%) was larger than 3 cm. Moderate differentiated tumor was the most common histology (*n* = 508, 51.8%), followed by poor differentiated tumor (*n* = 336, 34.3%) and well differentiated tumor (*n* = 136, 13.9%). According to the International tumor-node-metastasis-classification (American Joint Committee on Cancer/Union for International Cancer Control), 256 (26.1%), 360 (36.7%), 248 (25.3%) and 118 (12.0%) of colorectal cancer were classified as TNM stage I, II, III and IV respectively. The 10-gene panel test found that 161 (16.4%) tumors were MSI-H while 819 (83.6%) were MSS. Mutated KRAS, BRAF and PIK3CA was found in 330 (33.7%), 171 (17.4%) and 182 (18.6%) tumors, while wild-type of KRAS, BRAF and PIK3CA was found in 650 (66.3%), 809 (82.6%) and 798 (81.4%) tumors respectively.

**Table 1:** Statistical results of NDRG4 immunohistochemical staining

Variable	*n*	NDRG4 staining		*P*
		Negative (−)	Positive (+)	
	980	581	399	
**Sex**				0.766*
Male	520	306	214	
Female	460	275	185	
**Age at diagnosis**				0.083*
≤ 60	562	320	242	
>60	418	261	157	
**Tumor location**				0.791†
Right	221	132	89	
Left	280	170	110	
Rectum	479	279	200	
**Tumor size**				0.687*
≤3.0 cm	225	136	89	
>3.0 cm	755	445	310	
**Differentiation status**				0.001†
Well	136	65	71	
Moderate	508	302	206	
Poor	336	234	122	
**Depth of invasion**				0.007†
T_1_	125	60	65	
T_2_	232	132	100	
T_3_	496	302	194	
T_4_	127	87	40	
**Node metastasis**				0.004*
Absent(N0)	616	344	272	
Present(N1-3)	364	237	127	
**Distant metastasis**				0.001*
Absent(M0)	862	495	367	
Present(M1)	118	86	32	
**TNM stage**				0.001†
I	256	131	125	
II	360	223	137	
III	246	151	95	
IV	118	86	32	
**MSI**				0.330*
MSS	819	480	339	
MSI-H	161	101	60	
**KRAS mutation**				0.082*
(−)	650	398	252	
(+)	330	183	147	
**BRAF mutation**				0.069*
(−)	809	469	340	
(+)	171	112	59	
**PIK3CA mutation**				< 0.001*
(−)	798	452	346	
(+)	182	129	53	

* *P* value when expression levels were compared using Mann Whitney test.

† *P* value when expression levels were compared using Kruskal Wallis test.

### NDRG4 and p-AKT expression detected in clinical specimens

**Figure 1:**
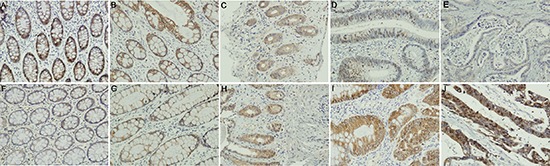
IHC staining of NDRG4 and p-AKT **(A–E)** NDRG4 staining, A normal epithelium, B chronic colonitis, C ulcerative colitis, D dysplasia, E cancer; **(F–J)** p-AKT staining, F normal epithelium, G chronic colonitis, H ulcerative colitis, I dysplasia, J cancer.

To understand the role of NDRG4 in colorectal cancer and to determine the potential functional mechanism, we began by investigating NDRG4 expression in clinical specimens and its association with clinicopathological characteristics as well as p-AKT. IHC assay results revealed that NDRG4 staining was mainly located in cytoplasma. Representative staining pattern of NDRG4 and p-AKT was showed in Figure 1. The positive ratio was significantly decreased from normal mucosa (89.7%, Figure 1A), chronic colitis (75.8%, Figure 1B), ulcerative colitis (71.8%, Figure 1C), atypical hyperplasia (58.8%, Figure 1D) to tumor (40.7%, Figure 1E) indicating its tumor suppressive role during the transition from normal mucosa to cancer (*P* < 0.001). Whereas p-AKT protein expression pattern was opposite to NDRG4 with IHC figures showed in Figure 1F–1J. With respect to the association of NDRG4 with p-AKT staining, there was a significant negative correlation between NDRG4 and p-AKT staining both in the retrospective (*r* = −0.409, *P* < 0.001) and prospective study cohorts (*r* = −0.140, *P* = 0.018), indicating the potential interaction of the two proteins. To detect the role of NDRG4 in colorectal cancer, we next examined the association of NDRG4 with clinicopathological characteristics in both study cohorts. Statistical analysis results (Table 1) showed that NDRG4 positive staining was significantly associated with tumor well differentiation (*P* = 0.001), little invasiveness (*P* = 0.007), absent node metastasis (*P* = 0.004), absent distant metastasis (*P* = 0.001) and low TNM stage (*P* = 0.001), indicating NDRG4 might play tumor suppressive role in colorectal cancer by regulating tumor differentiation, invasion and metastasis. Moreover, NDRG4 positive staining was also more likely to be detected in tumors with wild-type of PI3KCA (*P* < 0.001).

### Retrospective study

**Figure 2:**
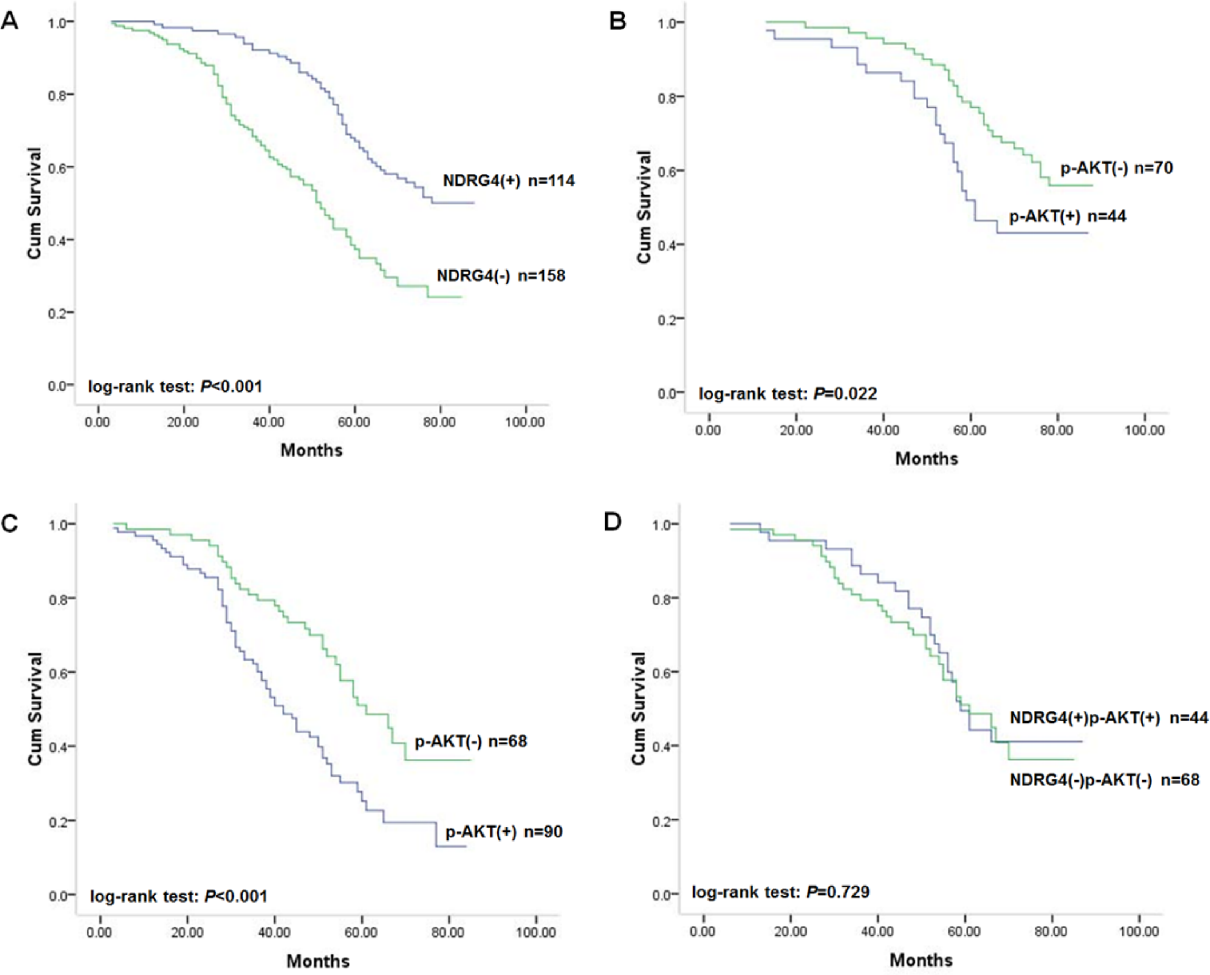
Kaplan-Meier survival curves of patients in the retrospective study cohort **(A)** Correlation of NDRG4 staining with overall survival. **(B)** Correlation of p-AKT staining with overall survival among patients with tumors with positive NDRG4 staining. **(C)** Correlation of p-AKT staining with overall survival among patients with tumors with negative NDRG4 staining. **(D)** Survival curves for patients with aberrant NDRG4 and p-AKT coexpression.

Given the strong association between NDRG4 and colorectal cancer differentiation, invasion and metastasis, we further investigate its prognostic role utilized the cut-off point of positive and negative staining of NDRG4. In the retrospective data set including 272 patients, 148 (54.4%) patients were dead at the end of follow-up. Survival analysis by Kaplan-Meier estimate and log-rank test revealed that the presence of NDRG4 positive staining was significantly associated with favorable outcome (log-rank test: *P* < 0.001; Figure 2A). The median survival time of patients with negative (−) staining of NDRG4 was 52 months (95% CI: 47.3–56.7), while that of patients with positive (+) staining of NDRG4 cannot be estimated due to better survival. Patients with negative NDRG4 staining tended to have a higher risk of death, with an unadjusted hazard ratio (HR) of 2.18 (95% CI: 1.11–4.33; *P* < 0.001). In addition, differentiation status, lymph node metastasis, TNM stage, KRAS, BRAF, PIK3CA mutations and MSI were also found to be associated with prognosis. However, sex, age, tumor location, tumor size or vascular invasion had no prognostic value (Table 2). Starting from these covariates, we constructed a multivariate prognostic model that considered the significant risk factors by using a stepwise selection procedure. Multivariate Cox regression adjusted for sex, age, differentiation status, TNM stage, KRAS, BRAF and PIK3CA mutations and MSI showed that negative NDRG4 staining was significantly and independently associated with poor overall survival, with an adjusted HR of 2.35 (95% CI: 1.15–4.61; *P* < 0.001). Thus, NDRG4 staining constitutes a powerful prognostic biomarker independent of adjusted clinical variables in retrospective cohort (Table 2).

**Table 2:** Association of molecule and clinical factors with overall survival in retrospective study

	Unadjusted HR* (95% CI)	*P*	Adjusted HR† (95% CI)	*P*
NDRG4	2.18 (1.11–4.33)	< 0.001	2.35 (1.15–4.61)	< 0.001
Sex	0.73 (0.49–1.09)	0.128	0.82 (0.52–1.29)	0.392
Age at diagnosis	1.12 (0.81–1.57)	0.490	1.13 (0.80–1.60)	0.487
Tumor location	1.29 (0.86–1.96)	0.423	1.17 (0.67–2.02)	0.582
Tumor size	1.69 (0.83–3.72)	0.056	1.61 (0.73–3.54)	0.235
Differentiation	2.33 (1.40–3.87)	0.001	1.24 (0.69–2.25)	0.474
Vascular invasion	1.91 (0.97–3.77)	0.061	0.63 (0.28–1.41)	0.260
TNM stage	5.76 (3.06–10.83)	< 0.001	3.90 (1.57–9.70)	0.003
MSI	1.88 (1.36–2.59)	< 0.001	1.58 (1.11–2.24)	0.011
KRAS mutation	1.56 (1.14–2.14)	0.006	1.46 (1.04–2.04)	0.028
BRAF mutation	1.75 (1.28–2.39)	< 0.001	1.56 (1.12–2.16)	0.008
PIK3CA mutation	1.82 (1.31–2.52)	< 0.001	1.65 (1.17–2.33)	0.004

* Hazard ratios in univariate models

† Hazard ratios in multivariable models

Abbreviations: HR, hazard ratio; 95% CI, 95% confidence interval.

As NDRG4 staining was found to be negatively correlated with that of p-AKT, in an attempt to investigate the prognostic power of NDRG4 according to p-AKT, we subsequently assigned patients into subgroups. In positive NDRG4 staining subgroup, patients with colorectal cancer of negative p-AKT staining had favorable survival (Figure 2B). While in NDRG4 negative staing subgroup, colorectal cancer of positive p-AKT staining had poor survival (Figure 2C). However, patients with colorectal cancer of both positive staining of NDRG4 and p-AKT was not significantly different from those with both negative staining in outcomes (Figure 2D). These results revealed that the prognostic value of NDRG4 on clinical outcome was stratified by p-AKT.

### Prospective cohort study

As it was proved that the cut-off point of NDRG4 staining utilized in retrospective study could effectively stratify colorectal cancer prognosis, we further recruited a prospective study cohort including 708 patients to validate its predictive role. The protein expression levels were comparable in both study cohorts for NDRG4 (positive: 43.5% v 40.3%) and p-AKT (positive: 51.1% v 53.0%); while 391 (55.2%) patients in prospective study cohort were dead at the time of analysis, compared with 148 (54.4%) in retrospective cohort, indicating. These results confirmed NDRG4 expression pattern and indicated the similarity of the two data sets in protein staining pattern and clinicopathological characteristics.

**Figure 3:**
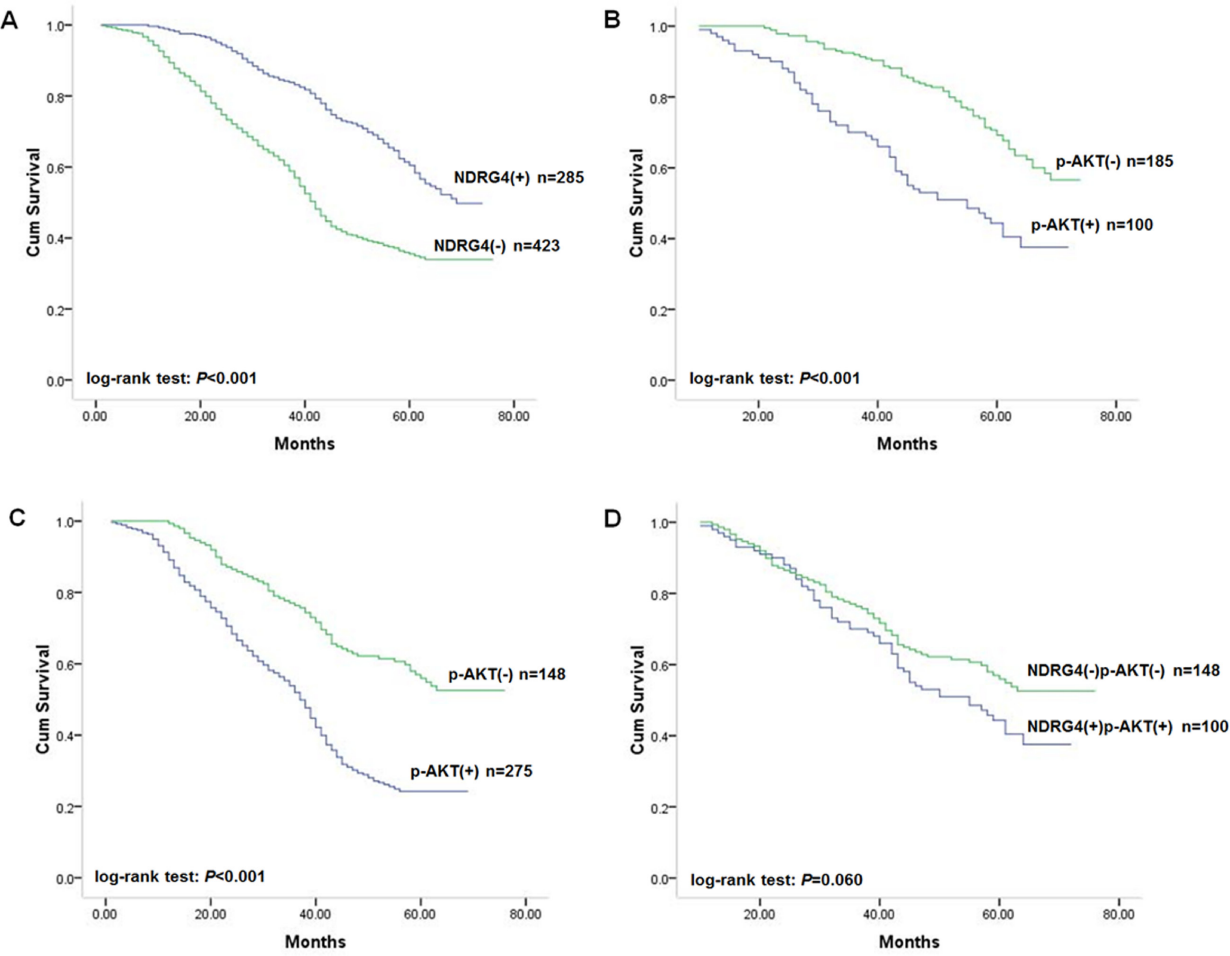
Kaplan-Meier survival curves of patients in the prospective study cohort **(A)** Correlation of NDRG4 staining with overall survival. **(B)** Correlation of p-AKT staining with overall survival among patients with tumors with positive NDRG4 staining. **(C)** Correlation of p-AKT staining with overall survival among patients with tumors with negative NDRG4 staining. **(D)** Survival curves for patients with aberrant NDRG4 and p-AKT coexpression.

Kaplan-Meier analysis and log-rank test found that NDRG4 negative staining was significantly associated with poor overall survival of patients (Figure 3A), with an unadjusted HR of 2.05 (95% CI: 1.12–4.35; *P* < 0.001). This finding further strengthened the role of NDRG4 as a prognostic marker in colorectal cancer. With respect to clinicopathological characteristics, differentiation status, vascular invasion, lymph node metastasis and TNM stage were also associated with prognosis, which was similar to results in retrospective cohort indicating the homogeneity of the two study cohorts (Table 3). Univariate Cox regression analysis was performed accordting to retrospective study to assess whether NDRG4 was an independent prognostic predictor of survival. Results proved that negative NDRG4 staining was a significant independent predictor of poor survival, with an adjusted HR for of 2.13 (95% CI: 1.16–4.53; *P* < 0.001). These results were consistent with that in retrospective cohort study (Table 3).

**Table 3:** Association of molecular and clinical factors with overall survival in prospective study

	Unadjusted HR* (95% CI)	*P*	Adjusted HR† (95% CI)	*P*
NDRG4	2.05 (1.12–4.35)	< 0.001	2.13 (1.16–4.53)	< 0.001
p-AKT	1.84 (1.06–3.87)	< 0.001	2.21 (1.26–4.95)	< 0.001
Sex	1.09 (0.91–1.29)	0.357	1.07 (0.90–1.28)	0.426
Age at diagnosis	0.97 (0.82–1.16)	0.768	1.01 (0.85–1.21)	0.882
Tumor location	1.12 (0.73–1.64)	0.636	1.14 (0.75–2.11)	0.583
Tumor size	1.46 (0.71–3.29)	0.074	1.52 (0.83–3.76)	0.374
Differentiation	2.60 (2.01–3.37)	< 0.001	1.45 (1.09–1.92)	0.011
Vascular invasion	2.28 (1.63–3.21)	< 0.001	1.44 (0.89–1.92)	0.064
TNM stage	3.78 (2.73–5.23)	< 0.001	2.35 (1.56–3.52)	< 0.001
MSI	1.90 (1.35–2.68)	< 0.001	1.57 (1.08–2.27)	0.018
KRAS mutation	1.51 (1.08–2.11)	0.016	1.44 (1.01–2.07)	0.045
BRAF mutation	1.65 (1.18–2.30)	0.003	1.49 (1.05–2.11)	0.026
PIK3CA mutation	1.83 (1.29–2.59)	0.001	1.64 (1.13–2.36)	0.008

* Hazard ratios in univariate models

† Hazard ratios in multivariable models

Abbreviations: HR, hazard ratio; 95% CI, 95% confidence interval.

Given the retrospective study found that the prognostic role of NDRG4 was modified by p-AKT, we then assigned patients into subgroups by combined NDRG4 and p-AKT staining to test the accordance in prospective study cohort. Kaplan-Meier analysis results showed that negative p-AKT staining was associated with favorable survival in both NDRG4 positive and negative staining group (Figure 3B, 3C), whereas no prognostic difference was detected between patients with both positive and both negative staining of the two protein (Figure 3D). In multivariate analysis adjusted for factors including sex, age, differentiation status, TNM stage, KRAS, BRAF and PIK3CA mutations and MSI, coexpression of NDRG4 positive and p-AKT negative staining was significantly associated with favorable outcome, whereas the opposite expression pattern was associated with poor survival (Table 4). These results were consistent with that in retrospective cohort, and indicated that NDRG4 was an independent prognostic predictor which could be stratified according to p-AKT.

**Table 4:** Multivariate analysis of patient survival by combined NDRG4 and p-AKT

Combined NDRG4/p-AKT	Retrospective cohort			Prospective cohort		
	*n*	Unadjusted HR (95% CI)	Adjusted HR (95% CI)	*n*	Unadjusted HR (95% CI)	Adjusted HR (95% CI)
NDRG4 + p-AKT−	70	1.00 (reference)	1.00 (reference)	185	1.00 (reference)	1.00 (reference)
NDRG4 + p-AKT+	44	1.45 (1.04–3.79)	1.62 (1.08–4.21)	100	1.42 (1.08–2.96)	1.57 (1.09–3.86)
NDRG4 − p-AKT−	68	1.51 (1.04–3.45)	1.76 (1.13–3.43)	148	1.39 (1.09–3.21)	1.62 (1.14–3.58)
NDRG4 − p-AKT+	90	2.12 (1.14–4.16)	2.43 (1.16–4.26)	275	2.06 (1.11–4.35)	2.29 (1.15–4.18)

### Effect of NDRG4 overexpression on PI3K-AKT activity

**Figure 4:**
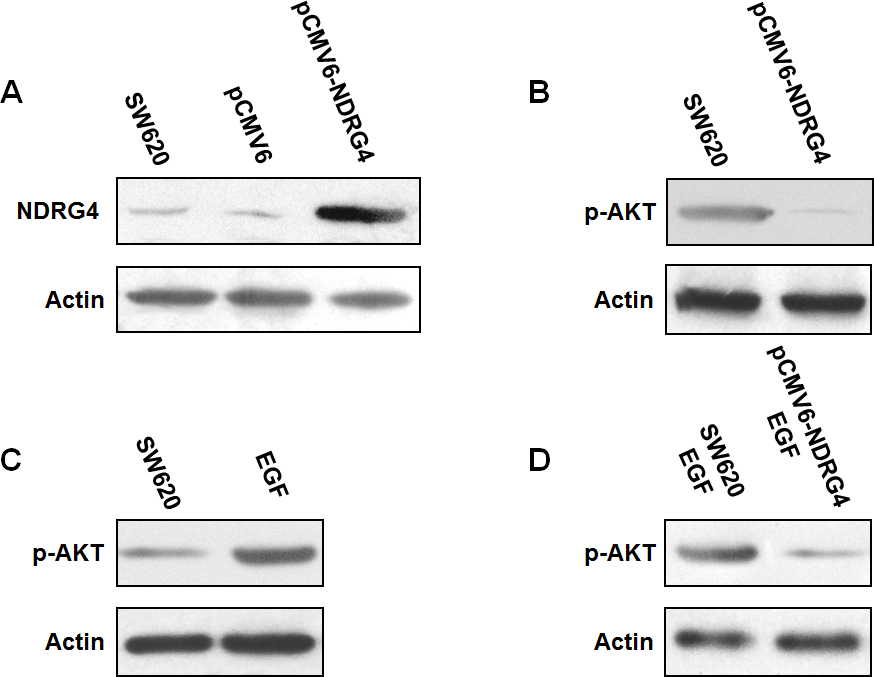
NDRG4 overexpression in SW620 cells reduced PI3K-AKT activation **(A)** Transfection of pCMV6-NDRG4 significantly increased NDRG4 expression. **(B)** Overexpression of NDRG4 decreased p-AKT expression. **(C)** EGF increased p-AKT expression. **(D)** NDRG4 overexpression suppressed p-AKT even after EGF stimulation.

As it was proved in clinical specimens that NDRG4 staining negatively correlated with p-AKT, which could stratify the prognostic role of NDRG4, indicating the potential interaction between NDRG4 and p-AKT. Considering the phosphorylation n of AKT is the key regulatory step of PI3K-AKT activation. We speculated that NDRG4 might play its tumor suppressive role through suppression of PI3K-AKT. To confirm this inhibitory effect of NDRG4 on PI3K-AKT activity, we firstly examined whether transfection of pCMV6-NDRG4 could increase NDRG4 expression in tumor cells. As shown in Figure 4A, transfection of pCMV6-NDRG4 in SW620 cells significantly increased NDRG4 expression compared with control cells. Then, we examined the effect of NDRG4 overexpression on p-AKT. Results showed that overexpression of NDRG4 considerably decreased p-AKT expression compared with control cells (Figure 4B). We next investigated the effect of NDRG4 overexpression on p-AKT in response to Epidermal Growth Factor (EGF) stimulation. It was found that the treatment with 5 nM PMA could lead to significant increase of p-AKT expression (Figure 4C). Whereas NDRG4 overexpression strongly suppressed p-AKT even after PMA stimulation (Figure 4D). These results indicated that NDRG4 could effectively suppress PI3K-AKT activity in colorectal cancer.

## DISCUSSION

We conducted the present study to determine NDRG4 protein expression pattern and its association with colorectal cancer carcinogenesis, progression, prognosis and PI3K-AKT activity. Results showed a statistically significant decrease of NDRG4 expression from normal mucosa, chronic colitis, ulcerative colitis, atypical hyperplasia to colorectal cancer. Notably, the positive ratio of NDRG4 in chronic colitis (75.8%) and ulcerative colitis (71.8%) was significantly decreased compared with that in normal mucosa (89.7%), indicating NDRG4 protein expression was associated with inflammation. In addition, the positive ratio of NDRG4 in atypical hyperplasia (58.8%) was lower than that in chronic colitis and ulcerative colitis, suggesting NDRG4 was related to early events of carcinogenesis. Whereas the expression pattern of p-AKT was opposite to NDRG4 among the same clinical specimens. In addition, statistical analysis revealed that p-AKT staining was negatively correlated with NDRG4. As long-standing inflammation was a hallmark of neoplastic transformation of epithelial cells in colorectal cancer [27–31]. These results suggested for the first time that NDRG4 might play a tumor suppressive role during colorectal cancer carcinogenesis, and have negative interaction with PI3K-AKT activity.

To clarify the role of NDRG4 in colorectal cancer, we investigated the association of NDRG4 staining with clinicopathological characteristics in both study cohorts. Results proved that NDRG4 positive staining was significantly associated with well differentiation, little invasiveness, absent node metastasis, absent distant metastasis and low TNM stage. In addition, NDRG4 positive staining was also more likely to be detected in tumors with wild-type of PI3KCA. This result together with the negative correlation between NDRG4 and p-AKT indicated NDRG4 might negatively interact with PI3K-AKT in colorectal cancer differentiation, invasion and metastasis possibly. This is the first direct evidence of the association between NDRG4 and clinicopathological characteristics of clinical colorectal cancer.

Given the close association found between NDRG4 and colorectal cancer progression, we further investigated its prognostic role in two separate cohorts. Results showed that positive staining of NDRG4 was independently associated with favorable outcome of patients in retrospective cohort, which was confirmed in independent prospective validation cohort. The prognostic value of NDRG4 was statistically significant in not only univariate analysis but also multivariate analysis adjusted for characteristics and other related molecular variables including KRAS, BRAF and PIK3CA mutations and MSI, which indicated NDRG4 protein expression may be a promising marker to predict prognosis of colorectal cancer. These results above were the first to address the prognostic role of NDRG4 in colorectal cancer. As p-AKT staining was proved to be negatively correlated with NDRG4, we assigned patients into subgroups according both NDRG4 and p-AKT staining to investigate whether the prognostic role of NDRG4 could be modified by p-AKT. Strikingly, among NDRG4 positive tumors, tumors with negative p-AKT staining showed significant favorable outcome, indicating the interaction of PI3K-AKT activity and NDRG4 might attenuated malignant biological behavior and improve subsequent prognosis. While tumors with positive p-AKT staining showed significant poor survival among NDRG4 negative ones, indicating the activation of PI3K-AKT activity without potential NDRG4 suppression might aggravated tumor malignant biological behavior and subsequent prognosis. Intriguingly, colorectal cancer with both positive NDRG4 and p-AKT staining showed no prognostic difference compared with those with both negative staining of NDRG4 and p-AKT. This could be possibly attributed to the aberrant regulation of PI3K-AKT activity by NDRG4, which accommodated tumor malignant biological behavior and subsequently attenuated the prognostic role of NDRG4. These results in line indicated that the prognostic role of NDRG4 in colorectal cancer was dependent on PI3K-AKT activity, which also further confirmed the interaction between NDRG4 and PI3K-AKT.

In the light of the strong association between NDRG4 and p-AKT found above including opposite expression pattern during carcinogenesis, negative correlation in clinical samples and stratified prognostic value of NDRG4 by p-AKT. Only demonstrate NDRG4 has the ability to suppress p-AKT can confirm NDRG4 plays its tumor suppressive role in carcinogenesis, progression and prognosis through the suppression of PI3K-AKT activity. For this purpose, we firstly transfected pCMV6-NDRG4 into tumor cells, which was proved to could increase NDRG4 expression. Western blot showed that overexpression of NDRG4 considerably decreased p-AKT expression even after EGF stimulation, which could lead to increase of p-AKT. These results together proved that NDRG4 could effectively suppress PI3K-AKT activity in colorectal cancer. Considering the activation of PI3K-AKT can lead to neoplastic transformation, tumor cell proliferation, invasion, angiogenesis and metastasis in colorectal cancer, it is rational that NDRG4 plays its tumor suppressive role in carcinogenesis, progression and prognosis of colorectal cancer at least partly by suppression of PI3K-AKT activity [32–35].

To our knowledge, NDRG4 was mainly investigated in central nerve system tumors before [6, 36, 37]. While it was found that NDRG2 and NDRG4 had opposing protein expression patterns when comparing normal brain tissue to glioblastoma tissue, suggesting the opposite function of NDRG4 to NDRG2, although NDRG4 is most similar to NDRG2 within the NDRG family [36, 38]. However, in contrast, the present study together with our previous work indicated that NDRG4 and NDRG2 protein expression was both decreased in colorectal cancer and similar had association with clinicopathological characteristics and prognosis [14, 39]. These findings in line with recent study which found NDRG4 promoter methylation was increased in colorectal cancer and NDRG4 overexpression could suppress colony formation, cell proliferation and invasion, indicated that NDRG4 might play the tumor suppressive role in colorectal cancer [11].

In addition, previous investigations also suggested that PI3K-AKT could support MYC activity by blocking its degradation in human malignancy [40, 41]. As downstream regulated by MYC, the expression of NDRG4 was transcriptionally repressed by it. It is therefore intriguing to speculate NDRG4 might be the central molecule participating in a more complex negative feedback loop in the regulation of PI3K-AKT activity. In this concept, NDRG4 is the key role to transfer its repression effect on PI3K-AKT activity. In normal mucosa, activate NDRG4 results in PI3K-AKT suppression, the following MYC inhibition leads to further NDRG4 expression. Then, the result of this negative regulation feedback loop is NDRG4 activation and consequent PI3K-AKT suppression. While in CRC carcinogenesis, the inhibition of NDRG4 causes the activation of PI3K-AKT, which further activates Myc and consequent NDRG4 suppression. Under this circumstance, the result of this negative regulation feedback loop is NDRG4 suppression and consequent PI3K-AKT activation.

Our study has several strengths. It included a retrospective hospital-based study cohort to explore the prognostic role of NDRG4 and its association with PI3K-AKT activity, as well as a prospective population-based study cohort for validation. The sample size was large and homogeneous, with adequate follow-up time and intimate information on clinicopathological characteristics. Traditionally, protein staining was used for diagnosis because the mRNA amount did not always correlate with protein level. However, scoring the staining can present problems for any immunohistochemistry-based detection. Here, we utilized an immunoreactivity score system with all of the cores on the slides viewed by two separate pathologists under a double-blind condition with rigorous comparison to internal controls. In addition, the results obtained in retrospective study cohort were also validated by that in prospective cohort, indicating the reliability of our results. To avoid protein expression being affected by preoperative neoadjuvant chemotherapy, we limited the cohort to patients who underwent surgical resection before the year 2006, before neoadjuvant chemotherapy were routinely used for CRC in the two medical centers. Thus, we were able to detect the independent predictive effect of NDRG4 on prognosis as well as its association with PI3K-AKT activity effectually and efficiently. Our current study comprehensively evaluated the association of NDRG4 with PI3K-AKT activity in CRC carcinogenesis and prognosis. The results provided the first evidence for NDRG4 to determine CRC carcinogenesis and prognosis by suppression of PI3K-AKT. These findings could also contribute to an accurate prediction of the CRC prognosis by the combination of NDRG4 and PI3K-AKT activity. Consequently, clinicians could suggest tailored treatments for individual patient, thus preventing patients from receiving excessive or insufficient adjuvant treatments, both of which are harmful. In addition, our results could also provide important clues in understanding how the PI3K-AKT signal is regulated by Myc and NDRG4 in tumor carcinogenesis and progression. In addition, we also investigated critical molecular events such as KRAS, BRAF and PIK3CA mutations and MSI, all of which have been associated with colorectal cancer prognosis to justify the independent prognostic role of NDRG4.

In conclusion, to our knowledge, this is the first study to describe the function of NDRG4 protein in carcinogenesis, progression and prognosis of colorectal cancer. Our study also showed for the first time that NDRG4 may play its tumor suppressive role through attenuation of PI3K-AKT activity. Our retrospective and prospective investigation indicated for the first time that NDRG4 protein could be an independent prognostic predictor to identify high risk colorectal cancer patients.

## MATERIALS AND METHODS

### Patients and specimens

This study was approved by the ethics committee of the Fourth Military Medical University. All patients recruited had provided full consent. The study population involved a hospital-based retrospective training cohort including 272 patients and a population-based prospective validation cohort including 708 patients, which have been described in our previous work. The hospital-based retrospective cohort included 272 patients who were randomly selected from patients consecutively diagnosed with colorectal cancer in Xijing Hospital, Fourth Military Medical University between January 2003 and December 2006. The population-based prospective cohort consisted of 681 patients with diagnosed colorectal cancer from January 2004 to December 2005, who were randomly selected from the Colorectal Cancer Registry, Tianjin Union Medicine Center, which serves a population of approximately 1.43 million.

All participating patients had given full informed consent. Patients with the following criteria were subsequently excluded: received treatment prior to surgery including neoadjuvant chemotherapy; harvested insufficient specimens for tissue microarray preparation; diagnosed as gastrointestinal stromal tumor or lymphoma; diagnosed with additional cancers; refused consent. All of these clinical cancerous specimens were collected by surgery or endoscopy. All of the specimens had been histologically diagnosed by Department of Pathology, Fourth Military Medical University. Patients' clinicopathological information and follow-up data of the remaining patients were prospectively entered into a database, which was under a close follow-up scheme and updated with respect to survival status every three months. Death of participants was ascertained by reporting from the family and verified by a review of public records. All of the fresh tissues were obtained within 10 minutes after surgical removal. Specimens were fixed in 10% formaldehyde and embedded in paraffin for histological sections. For both study cohorts, study physicians who reviewed all the records of colon cancer and recorded data the database were completely blind to exposure data. In addition, we also recruited pathologically confirmed normal mucosa (*n* = 58), chronic colitis (*n* = 62), ulcerative colitis (*n* = 71) and colorectal atypical hyperplasia (*n* = 68) to investigate the protein variation during the transition from normal mucosa to cancer.

### Immunohistochemistry assay

Tissue specimens were made into tissue array as described before [42, 43]. All specimens were fixed in 10% formalin and embedded in paraffin wax. HE slides from all the recruited tumors were viewed under a light microscope, areas of representative tumor regions were marked by a pathologist. Corresponding paraffin-embedded gastric cancer tissue blocks were obtained. Two tissue cylinders with a diameter of 1.0 mm were punched from the marked areas of each donor block with a tissue arrayer and placed into a recipient paraffin block. The cylinder was transferred carefully with a forceps to a recipient metal paraffin block box that contained a thin layer of soft wax to fix the cylinders. After all cylinders were aligned in the box, the box was covered with a plastic cassette, then, liquid wax was poured gently into the box until it was full. The box was put on a hotplate for 1 minute to homogenize the wax. At that point, the box was removed from the hotplate and cooled to room temperature slowly. At last, 4 μm sections of the product tissue array blocks were transferred to glass slides.

Immunohistochemistry (IHC) assay was performed as described before. Briefly, all sections were deparaffinized in xylene and dehydrated through a gradient concentration of alcohol before endogenous peroxidase activity was blocked with 0.5% H_2_O_2_ in methanol for 10 min. After nonspecific binding was blocked, the slides were incubated with primary antibody, NDRG4 (Abnova, H00065009-M01) or phospho-AKT (Abcam, ab8932), overnight in a moist box. Biotinylated goat anti-mouse IgG (Sigma) or goat anti-rabbit IgG (Sigma) was incubated with the sections for 1 h at room temperature and detected with a streptavidin-peroxidase complex. The brown color indicative of peroxidase activity was developed by incubating with 0.1% 3, 3-diaminobenzidine (Sigma) in PBS with 0.05% H_2_O_2_ for 5 min at room temperature. Negative controls were performed by replacing the primary antibody with pre-immune serum. Positive controls were performed as suggested by the manufacturer. These controls were conducted in each run of immunohistochemistry assay.

### Evaluation of staining

Staining was scored independently by two pathologists blinded to the clinicopathological features and outcome. An immunoreactivity score (IRS) system, based on the proportion and intensity of positively stained cancer cells, was applied [44]. The staining was evaluated by scanning the entire tissue specimen under low magnification (×40) and then confirming under high magnification (×200 and × 400). The extensional standard is as follows: ① number of positively stained cells ≤ 5% scored 0; 6%–25% scored 1; 26%–50% scored 2; 51%–75% scored 3; > 75% scored 4, ② intensity of stain: colorless scored 0; pallide-flavens scored 1; yellow scored 2; brown scored 3. We multiplied ① and ②, and the staining grade was stratified as absent (0 score), weak (1–4 score), moderate (5–8 score) and strong (9–12 score). All of the stained slides were interpreted by another pathologist unaware of the other data. Specimens were rescored if the difference of scores from the two pathologists was more than 3. The concordance of NDRG4 scoring between the two observers was 0.92 (j = 0.62; *P* < 0.001) and for p-AKT was 0.95 (j = 0.66; *P* < 0.001), indicating substantial agreement. Tumors with weak, moderate or strong immunostaining intensity were classified as positive (+) staining, whereas tumors with absent immunostaining were classified as negative (–) staining.

### DNA extraction, microsatellite instability (MSI), pyrosequencing of KRAS, BRAF and PIK3CA analysis

DNA was extracted from paraffin embedded tissue, MSI status was determined via testing on a 10-gene panel in tumor DNA using 10 microsatellite markers (BAT25, BAT26, BAT40, MYCL, D5S346, D17S250, ACTC, D18S55, D10S197, and BAT34C4) as described in previous study [45]. In brief, tumors with MSI-high/microsatellite stability (MSI-H) was defined if instability was observed for ≥ 30% of markers, while and MSI-low/microsatellite stability (MSS) was defined if instability was observed for < 30% of the markers. And we also performed PCR and pyrosequencing targeted for KRAS (codons 12 and 13), BRAF (codon 600) and PIK3CA (exons 9 and 20) [46–48].

### Cell culture, transfection and western blot

Human colorectal cancer cell lines SW620 was cultured in L-15 culture medium (Invitrogen) supplemented with 10% fetal calf serum [49]. Full length NDRG4 cDNA was cloned into a pCMV6-Neo vector to create pCMV6-NDRG4. SW620 cells were transfected with pCMV6-NDRG4 or empty pCMV6 by Nucleofector Kit V (Amaxa Biosystems) according to the manufacturer's protocol. Western blot was performed by protocol described before by use of NDRG4 (Abnova, H00065009-M01) and p-AKT (Abcam, ab8932) primary antibody.

### Statistical analysis

Statistical analysis was conducted using SPSS statistical software (version 13.0). Spearman's rank test was used to assess the correlation between the expression of NDRG4 and p-AKT. Survival curves were estimated using the Kaplan-Meier method, and differences in survival distributions were evaluated by the log-rank test. Cox's proportional hazards modeling of factors potentially related to survival was performed to calculate hazard ratios (HR) and identify which factors might have a significant influence on survival. The age of diagnosis was used as a continuous variable when adjusting for potential confounding, while other covariates were used as categorical variables. Differences with a *P* value of 0.05 or less were considered to be statistically significant, and all *P* values were determined from two-sided tests.
